# Estimating epidemic exponential growth rate and basic reproduction number

**DOI:** 10.1016/j.idm.2019.12.009

**Published:** 2020-01-08

**Authors:** Junling Ma

**Affiliations:** Department of Mathematics and Statistics, University of Victoria, Victoria, BC, V8W 2Y2, Canada

**Keywords:** Epidemic curve, Exponential growth rate, Maximum likelihood estimation, Phenomenological models

## Abstract

The initial exponential growth rate of an epidemic is an important measure of the severeness of the epidemic, and is also closely related to the basic reproduction number. Estimating the growth rate from the epidemic curve can be a challenge, because of its decays with time. For fast epidemics, the estimation is subject to over-fitting due to the limited number of data points available, which also limits our choice of models for the epidemic curve. We discuss the estimation of the growth rate using maximum likelihood method and simple models.

This is a series of lecture notes for a summer school in Shanxi University, China in 2019. The contents are based on Ma et al. (Ma, Dushoff, Bolker, & Earn, 2013). We will study the initial exponential growth rate of an epidemic in Section 1, the relationship between the exponential growth rate and the basic reproduction number in Section 2, an introduction to the least square estimation and its limitations in Section3, an introduction to the maximum likelihood estimation in Section 4, and the maximum likelihood estimation of the growth rate in Section 5.

## 1 Epidemic exponential growth rate

**Fig. 1:**
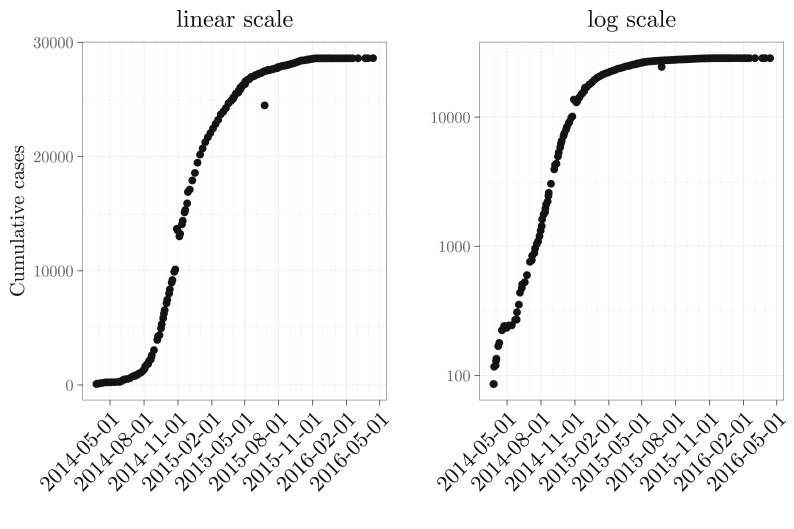
Cumulative Ebola cases during the 2014–16 western African Ebola outbreak, plotted in linear scale (left) and log-linear scale (right). Source: Center for Disease Control Ebola case counts (Center for Disease Control, 2016).

**Fig. 2:**
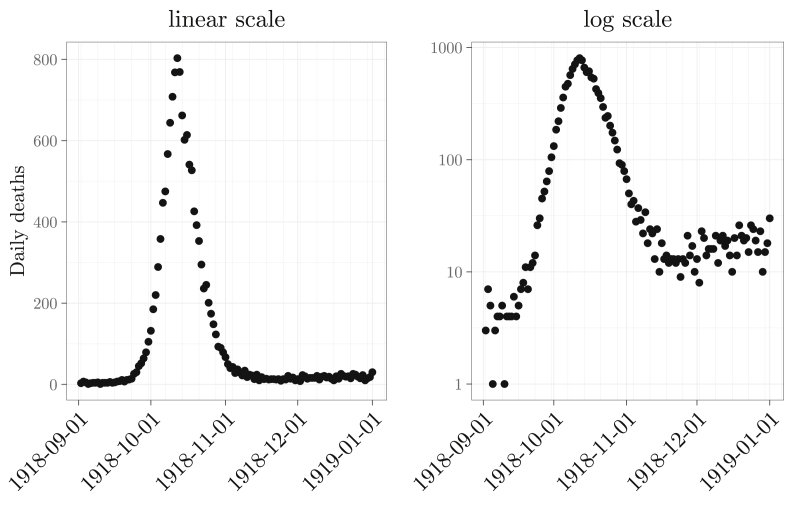
Weekly influenza mortality during the 1918 pandemic in Philadelphia, plotted in linear scale (left) and log-linear scale (right).

Epidemic curves are time series data of the number of cases per unit time. Common choices for the time unit include a day, a week, a month, etc. It is an important indication for the severeness of an epidemic as a function of time. For example, Fig. 1 shows the cumulative number of Ebola cases during the 2014–16 Ebola outbreak in western Africa. The cumulative cases during the initial growth phase form an approximately linear relationship with time in log-linear scale. Thus, in linear scale, the number of deaths increases exponentially with time. The mortality curve (the number of deaths per unit time) shows a similar pattern, as demonstrated by the daily influenza deaths in Philadelphia during the 1918 influenza pandemic shown in Fig. 2.

In fact, most epidemics grow approximately exponentially during the initial phase of an epidemic. This can be illustrated by the following examples.

Example 1 Consider the following Susceptible-Infectious-Recovered (SIR) model:

(1a) $$\frac{dS}{dt}=-\beta SI\;,$$

(1b) $$\frac{dI}{dt}=\beta SI-\gamma I\;,$$

(1c) $$\frac{dR}{dt}=\gamma I$$

where *S* is the fraction of susceptible individuals, *I* is the fraction of infectious individuals, and *R* is the fraction of recovered individuals; β is the transmission rate per infectious individual, and γ is the recovery rate, i.e., the infectious period is exponentially distributed with a mean 1/γ. Linearize about the disease-free equilibrium (DFE) (1,0,0),

(2) $$\frac{dI}{dt}\approx \left(\beta -\gamma \right)I.$$

Thus, if β−γ>0, then I(t) grows exponentially about the DFE. In addition, initially, S≈1, thus, the incidence rate (number of new cases per unit time) C=βSI also increases exponentially.

It is similar for an Susceptible-Exposed-Infectious-Recovered (SEIR) model, as illustrated by the following example.

Example 2 Lets consider an SEIR model:

(3a) $$\frac{dS}{dt}=-\beta SI,$$

(3b) $$\frac{dE}{dt}=\beta SI-\sigma E$$

(3c) $$\frac{dI}{dt}=\sigma E-\gamma I,$$

(3d) $$\frac{dR}{dt}=\gamma I,$$

where *E* is the fraction of latent individuals (infected but not infectious), σ the rate that latent individuals leaving the class, i.e; , the mean latent period is exponentially distributed with mean 1/σ; *S*, *I*, *R*, β and γ are similarly defined as in Example 1. Again, (1,0,0,0) is a disease free equilibrium representing a completely susceptible population. Linearize about this equilibrium, the equations for *E* and *I* are decoupled, and become

$$\frac{dE}{dt}=-\sigma E+\beta I,$$

$$\frac{dI}{dt}=\sigma E-\gamma I.$$

Note that the Jacobian matrix

$$J=\left[\begin{array}{cc} -\sigma & \beta \\ \sigma & -\gamma \end{array}\right]$$

has two real eigenvalues, namely,

$$\lambda_{1}=\frac{-\left(\sigma +\gamma \right)+\sqrt{{\left(\sigma -\gamma \right)}^{2}+4\sigma \beta }}{2},\;\lambda_{2}=\frac{-\left(\sigma +\gamma \right)-\sqrt{{\left(\sigma -\gamma \right)}^{2}+4\sigma \beta }}{2}.$$

Thus, about the DFE, the solution of the model is asymptotically exponential with a rate $\lambda_{1}$. Similar to Example 1, the incidence rate also grows exponentially initially.

In general, suppose the infection states of an individual can be characterized by the following vector $\left(\vec{S},\vec{I}\right)$, where $\vec{S}$ represents multiple susceptible states, and $\vec{I}$ represents multiple infectious (or latent) states. We also use $\vec{S}$ and $\vec{I}$ represent the number of individuals in each state. Also assume that the epidemic can be modeled by the following generic system

$$\frac{d}{dt}\vec{S}=f\left(\vec{S},\vec{I}\right),$$

$$\frac{d}{dt}\vec{I}=g\left(\vec{S},\vec{I}\right),$$

Assume that $g\left(\vec{S},0\right)=0$ for all $\vec{S}$; in addition, $\left({\vec{S}}_{0},\vec{0}\right)$ is a DFE, and the initial number of infectious individuals $\vec{I}\left(0\right)$ is very small, then, initially, the dynamics of *I* is governed by the following linearized system

$$\frac{d}{dt}\vec{I}=\frac{\partial g}{\partial \vec{I}}\left(S_{0},0\right)\vec{I}.$$

If the DEF is unstable, then I(t) grows asymptotically exponentially.

## 2 The exponential growth rate and the basic reproduction number

The exponential growth rate is, by itself, an important measure for the speed of spread of an infectious disease. It being zero is, like the basic reproduction number $R_{0}=1$, a disease threshold. The disease can invade a population if the growth rate is positive, and cannot invade (with a few initially infectious individuals) if it is negative. In fact, it can be used to infer $R_{0}$.There are two approaches to infer $R_{0}$ from the exponential growth rate, a parametric one, and a non-parametric one.

### 2.1 The parametric approach

For the parametric approach, we need an underlying model that gives both the growth rate and $R_{0}$.

Example 3 Consider the SIR model (1) in Example 1. Note that (1,0,0) is an disease free equilibrium, representing a completely susceptible population. As we discussed above, the exponential growth rate is λ=β−γ. Note that the basic reproduction number is $R_{0}=\beta /\gamma$ . If, for example, γ is estimated independently to λ, then,

$$R_{0}=\frac{\lambda }{\gamma }+1.$$

Lets look at a more complicated example.

Example 4 Lets consider the SEIR model (3) in Example 2. The basic reproduction number is $R_{0}=\beta /\gamma$. To link $R_{0}$ to the exponential growth rate

$$\lambda =\frac{-\left(\sigma +\gamma \right)+\sqrt{{\left(\sigma -\gamma \right)}^{2}+4\sigma \beta }}{2},$$

express β in terms of λ and substitute it into $R_{0}$, then

$$R_{0}=\frac{\left(\lambda +\sigma \right)\left(\lambda +\gamma \right)}{\sigma \gamma }.$$

Thus, if the mean infectious period 1/γ and the mean latent period 1/σ can be independently estimated on λ, then $R_{0}$ can be inferred from λ.

Typically, for an epidemic model that contains a single transmission rate β, if all other parameters can be estimated independently to the exponential growth rate λ, then λ determines β, and thus determines $R_{0}$.

### 2.2 The non-parametric approach

Models can be overly simplified for mathematical tractability. For example, Both the SIR model in Example 1 and the SEIR model in Example 2 assume exponentially distributed infectious period. However, the infectious period and the latent period are mostly likely not exponential. Wallinga and Lipsitch (Wallinga & Lipsitch, 2006) developed a non-parametric method to infer the basic reproduction number from the exponential growth rate without assuming a model.

Let η(a) be the probability that a random individual remain infectious *a* time units after being infected (i.e., *a* is the infection age); β(a) is the rate of transmission at the infection age *a*. Then,

τ(a)=η(a)β(a)

is the transmissibility of a random infectious individual at the infection age *a*, assuming that the whole population is susceptible. Thus,

$$R_{0}=\int_{0}^{\infty }\tau \left(a\right)da.$$

In addition, we assume that the population is randomly mixed, i.e., every pair of individuals have identical rate of contact. Let c(t)dt be the number of new infections during the time interval [t,t+dt], that is, c(t) is the incidence rate, and S(t) be the average susceptibility of the population, i.e., the expected susceptibility of a randomly selected individual. In addition, new infections at time *t* is the sum of all infections caused by infectious individuals infected *a* time unit ago (i.e., at time t−a) if they remain infectious at time *t* (with an infectious age *a*) and their contact is susceptible. That is,

$$c\left(t\right)=\int_{0}^{\infty }c\left(t-a\right)\tau \left(a\right)S\left(t\right)da,$$

and thus

$$c\left(t\right)=S\left(t\right)\int_{0}^{\infty }c\left(t-a\right)\eta \left(a\right)da.$$

To compute $R_{0}$, we need to normalize τ(a) as a probability density function,

$$w\left(a\right)=\frac{\tau \left(a\right)}{\int_{0}^{\infty }\tau \left(s\right)ds}=\frac{\tau \left(a\right)}{R_{0}}.$$

Note that w(a)da is the probability that a secondary infection occurs during the infection age interval [a,a+da]. That is, w(a) is the probability density function of the generation time, i.e., the time from being infected to generate a secondary infection. This generation time is also called the serial interval. With the serial interval distribution w(t),

(4) $$c\left(t\right)=R_{0}S\left(t\right)\int_{0}^{\infty }c\left(t-a\right)w\left(a\right)da.$$

This means that the c(t) is only determined by $R_{0}$, w(t) and S(t). At the beginning of an epidemic, where the epidemic grows exponentially (with an exponential growth rate λ), S(t)≈1 and $c\left(t\right)=c_{0}e^{\lambda t}$ where $c_{0}$ is the initial number of cases at time t=0. Thus,

$$e^{\lambda t}=R_{0}\int_{0}^{\infty }e^{\lambda \left(t-a\right)}w\left(a\right)da,$$

that is,

(5) $$R_{0}=\frac{1}{\int_{0}^{\infty }e^{-\lambda a}w\left(a\right)da}=\frac{1}{M\left(-\lambda \right)},$$

where $M\left(x\right)=\int_{0}^{\infty }e^{xa}w\left(a\right)da$ is the moment generating function of the serial time distribution w(a).

Equation (5) links the exponential growth rate to the basic reproduction number though the serial interval distribution only. That is, if we can estimate the serial interval distribution and the exponential growth rate independently, that we can infer the basic reproduction number.

Note that the serial interval distribution w(t) can be estimated independently to the exponential growth rate. For example, it can be estimated empirically using contact tracing. Alternatively, one can also assume an epidemic model. Here we discuss a few simple examples.

Example 5 Consider an SIR model. Let F(a) be the cumulative distribution function of the infectious period, and a constant transmission rate β. The probability that an infected individual remains infectious *a* time units after being infected is

η(a)=1−F(a),

and thus the transmissibility is

τ(a)=β[1−F(a)],

and the serial interval distribution is

$$w\left(a\right)=\frac{\tau \left(a\right)}{\int_{0}^{\infty }\tau \left(t\right)dt}=\frac{1-F\left(a\right)}{\int_{0}^{\infty }1-F\left(a\right)dt}=\frac{1-F\left(a\right)}{\mu },$$

where μ is the mean infectious period. For the special case that the infectious period is exponentially distributed with a rate γ, i.e., $F\left(a\right)=1-e^{-\gamma a}$, this model becomes Model (1). Then the density function of serial interval distribution is

$$w\left(a\right)=\frac{1-F\left(a\right)}{1/\gamma }=\gamma e^{-\gamma a}$$

which is identical to the density function of infectious period distribution. The moment generating function is

$$M\left(x\right)=\frac{\gamma }{\gamma -x},$$

Note that the exponential growth rate is λ=β−γ, then

$$R_{0}=\frac{1}{M\left(-\lambda \right)}=\frac{\gamma +\lambda }{\gamma }=\frac{\beta }{\gamma }.$$

Lets consider a more complex example with multiple infected states.

Example 6 Consider an SEIR model with a constant transmission rate β. Let F(a) and G(a) be the cumulative distribution functions of the infectious period and the latent period, respectively. Given the latent period $T_{L}=\ell \leq a$, the probability that an infectious individual is infectious *a* time units after being infected is 1−F(a−ℓ).Thus,

$$\eta \left(a\right)=\int_{0}^{a}1-F\left(a-\ell \right)dG\left(\ell \right).$$

Hence, the serial interval distribution is

$$w\left(a\right)=\frac{\int_{0}^{a}\left[1-F\left(a-\ell \right)\right]G’\left(\ell \right)d\ell }{\int_{0}^{\infty }\int_{0}^{a}\left[1-F\left(a-\ell \right)\right]G’\left(\ell \right)d\ell da}.$$

For the special case that the latent period is exponentially distributed with a rate σ (i.e., $F\left(a\right)=1-e^{-\gamma a}$) and the latent period is exponentially distributed with a rate σ (i.e., $G\left(a\right)=1-e^{-\sigma a}$), this model becomes Model (3), and

$$w\left(a\right)=\gamma \sigma e^{-\gamma a}\int_{0}^{a}e^{\left(\gamma -\sigma \right)s}ds=\left(\gamma e^{-\gamma a}\right)\text{*}\left(\sigma e^{-\sigma a}\right).$$

That is, if both distributions are exponential, the serial interval distribution is the convolution of the latent period distribution and the infectious period distribution. In this case, the basic reproduction number is

$$R_{0}=\frac{1}{M\left(-\lambda \right)}=\frac{1}{M_{I}\left(-\lambda \right)M_{L}\left(-\lambda \right)}=\frac{\left(\lambda +\gamma \right)\left(\lambda +\sigma \right)}{\gamma \sigma },$$

where $M_{I}\left(x\right)$ and $M_{L}\left(x\right)$ are the moment generating functions of the infectious period and latent period, respectively.

#### Remark

In Equation (4), $R\left(t\right)=R_{0}S\left(t\right)$ is the reproduction number, and thus this equation can be used to estimate the production number at any time *t* during the epidemic given the incidence curve c(t), namely,

$$R\left(t\right)=\frac{c\left(t\right)}{\int_{0}^{\infty }c\left(t-a\right)w\left(a\right)da}.$$

This is similar to, but different from, the nonparametric method developed by Wallingua and Teunis (Wallinga & Teunis, 2004).

## 3 Least squares estimation

The least squares method is one of the most commonly used methods for parameter estimation in mathematical biology. This method is in fact a mathematical method. For a family of curves $f\left(t;\vec{\theta }\right)$, where $\vec{\theta }\in R^{m}$ is a vector of parameters of the family, this method finds the curve $f\left(t;\hat{\theta }\right)$ in the family that minimizes the distance between the curve and a set of points ${\left\{\left(t_{i},x_{i}\right)\right\}}_{i=0}^{n-1}$. Let $\vec{x}=\left(x_{0},\ldots ,x_{n-1}\right)$, and $\vec{f}\left(\vec{\theta }\right)=\left(f\left(t_{0};\vec{\theta }\right),\ldots ,f\left(t_{n-1};\vec{\theta }\right)\right)$, and $\vec{x}$ be the Euclidean norm in $R^{n}$, then the mathematical formulation of the least squares method is

(6) $${\hat{\theta }=\underset{\vec{\theta }}{argmin}{\left\|\vec{f}\left(\vec{\theta }\right)-\vec{x}\right\|}^{2}=\underset{\vec{\theta }}{argmin}\overset{n-1}{\underset{i=0}{\sum }}\left[f\left(t_{i};\vec{\theta }\right)-x_{i}\right]}^{2},$$

where argmin gives the parameter $\vec{\theta }$ that minimizes the objective function. For our purpose, the observations ${\left\{\left(t_{i},x_{i}\right)\right\}}_{i=0}^{n-1}$ is the epidemic curve, i.e., $x_{0}$ is the number of initially observed cases, and $x_{i}$ is the number of new cases during the time interval $\left(t_{i-1},t_{1}\right]$. We aim to find an exponential function $f\left(t;c_{0},\lambda \right)=c_{0}e^{\lambda t}$ that minimizes its distance to the epidemic curve, i.e., the parameters $\theta =\left(c_{0},\lambda \right)$. There are two commonly use methods to estimate the exponential growth rate λ:

1. Nonlinear least square to fit to $f\left(t;c_{0},\lambda \right)=c_{0}e^{\lambda t}$ directly;
2. Linear least square to fit $\left\{\left(t_{i},\ln x_{i}\right)\right\}$ to $\ln\;f\left(t;c_{0},\lambda \right)=\ln c_{0}+\lambda t$.

The nonlinear least squares method does not have an analytic solution. Numerical optimization is needed to solve the minimization problem (6). The linear least square method has an analytic solution: Let $\ell_{0}=\text{ln}c_{0}$, then the least squares problem becomes

$$\left(\ell_{0},\lambda \right)=\underset{\left(\ell_{0},\lambda \right)}{argmin}\overset{n-1}{\underset{i=0}{\sum }}{\left(\ell_{0}+\lambda t_{i}-\text{ln}x_{i}\right)}^{2}.$$

The objective function is a quadratic function of $\ell_{0}$ and λ, thus, the minimum is achieved at $\left({\hat{\ell }}_{0},\hat{\lambda }\right)$ that satisfies

$$\frac{\partial s}{\partial \ell_{0}}{|}_{\overset{\ell_{0}={\hat{l}}_{0}}{\lambda =\hat{\lambda }}}=\overset{n-1}{\underset{i=0}{\sum }}2({\hat{\ell }}_{0}+\hat{\lambda }t_{i}-\ln x_{i})=0,$$

$$\frac{\partial s}{\partial \lambda }{|}_{\overset{\ell_{0}={\hat{l}}_{0}}{\lambda =\hat{\lambda }}}=\overset{n-1}{\underset{i=0}{\sum }}2t_{i}({\hat{\ell }}_{0}+\hat{\lambda }t_{i}-\ln x_{i})=0.$$

Let $\langle y_{i}\rangle =\frac{1}{n}\sum_{i=0}^{n-1}y_{i}$, which represents the average of any sequence ${\left\{y_{i}\right\}}_{i=0}^{n}$, then,

$$\left[\begin{array}{cc} 1 & \left\langle t_{i}\right\rangle \\ \left\langle t_{i}\right\rangle & \left\langle t_{i}^{2}\right\rangle \end{array}\right]\left[\begin{array}{c} {\hat{\ell }}_{0}\\ \hat{\lambda } \end{array}\right]=\left[\begin{array}{c} \left\langle \ln x_{i}\right\rangle \\ \left\langle t_{i}\;\ln x_{i}\right\rangle \end{array}\right],$$

and thus the best fit exponential growth rate ls

$$\hat{\lambda }=\frac{\left\langle t_{i}\;\ln x_{0}\rangle -\langle t_{i}\rangle \langle \ln x_{i}\right\rangle }{\langle t_{i}^{2}\rangle -\langle t_{i}\rangle^{2}}.$$

**Fig. 3:**
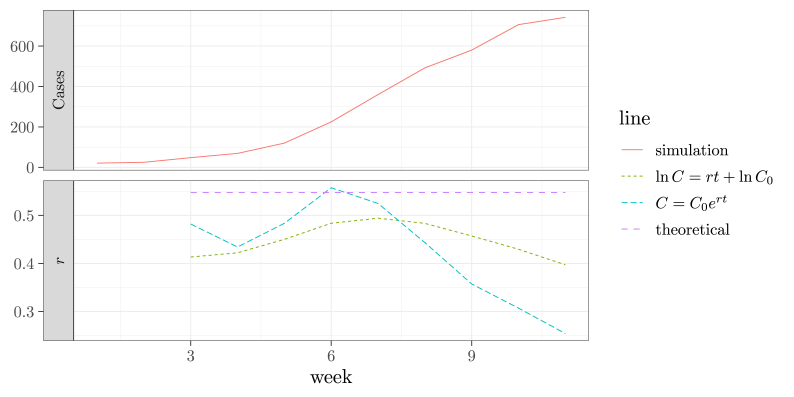
The simulated SEIR epidemic curve (upper) and the fitted exponential growth rate as a function of the end of the fitting window (lower). The epidemic curve is simulated stochastically from the SEIR model in Example 2 using the Gillespie method (Gillespie, 1976) with the parameters β=0.3, σ=1, γ=0.2, $E_{0}=10$, $S_{0}=9,990$. $I_{0}=R_{0}=0$. The rates have a time unit of a day. The daily cases are then aggregated by week. The data points are taken at times $t_{i}=i$, i=0,1,2,…13 weeks. The theoretical exponential growth rate is λ=0.547 per week.

Do these two methods yield the same answer? To compare, we simulate an epidemic curve of the stochastic SEIR model in Example 2, using the Gillespie method (Gillespie, 1976). The simulated daily cases (number of individuals showing symptom on a day) are then aggregated into weekly cases. Then, we use both methods to fit an exponential curve to the simulated epidemic curve. The simulated epidemic curve and the fitting results are shown in Fig. 3. This exercise illustrates a challenge of fitting an exponential model to an epidemic curve: how to determine the time period to fit the exponential model. The exponential growth rate of an SEIR model decreases with time as the susceptible population decreases. In Fig. 3, The epidemic curve peaks in week 13. We choose a sequence of nested fitting windows starting in the first week and ending in a week *w* for w=3,4,…,13. The SEIR model has an asymptotic exponential growth, so the fitted exponential growth rate is not monotonic near the beginning of the epidemic. For larger fitting windows, both methods give an exponential growth rate that decreases with the length of the fitting window. We need more data points to reduce the influence of the stochasticity. However, using more data points also risks of obtaining an estimate that deviates too much from the true exponential growth rate. There is no reliable method to choose a proper fitting window.

Fig. 3 also shows that the linear and nonlinear least squares methods may not yield the same estimate. This is because of a major limitation of both least squares methods: they implicitly assume that the deviations $\left|x_{i}-f\left(t_{i};\vec{\theta }\right)\right|$ carry identical weights. With the nonlinear method, later data points (at larger times) deviate more from the exponential curve than the earlier data points, because the exponential growth slows down with time. Thus, the method is more biased to the later data points. With the linear method, the deviations in $\text{ln}x_{i}$ are more even than in $x_{i}$, and thus the linear method is less biased to the later data points than the nonlinear method does.

The least squares method, as mentioned above, is a mathematical problem. It does not explicitly assume any error distributions, and thus cannot give us statistical information about the inference. For example, if we use two slightly different fitting windows and get two slightly different estimates, is the difference of the two estimates statistically significant? Such a question cannot easily be answered by the least squares method. Interestingly, the least squares methods make many implicit assumptions to the deviations. We have mentioned the implicit equal-weight assumption above. It also implicitly assumes that the order of the observations does not matter, and that positive and negative deviations are equivalent. Thus, they implicitly assume that the deviations are independently identically and symmetrically distributed. In statistics, the least squares method is commonly used in linear and nonlinear regression with an addition assumption that the errors are independently and identically normally distributed. However, these assumption on the errors may not be appropriate. For example, the new cases at time t+1 may be infected by those who are infected at time *t*. Thus, the number of new cases at different times may not be independent. Also, the number of cases is a counting variable, and thus its mean and variance may be closely related, meaning that the error may not be identically normally distributed. In the next section, we address some of these problems using the maximum likelihood method.

## 4 Maximum likelihood estimation

The maximum likelihood method is a commonly used statistical method for parameter inference; see, e.g., [(Bolker, 2008), p.170]. It relies on a “likelihood function” $L\left(\vec{\theta }\right)$ where $\vec{\theta }$ is the vector of parameters. The likelihood function is a function proportional to the conditional probability of observing the data points ${\left\{\left(t_{i},x_{i}\right)\right\}}_{i=0}^{n-1}$ given the parameters $\vec{\theta }$, i.e.,

$$L\left(\vec{\theta }\right)\propto P\left({\left\{\left(t_{i},d_{i}\right)\right\}}_{i=0}^{n-1}|\vec{\theta }\right).$$

We choose the parameter values that maximize the likelihood, i.e.,

$$\hat{\theta }=\underset{\vec{\theta }}{\text{argmin}}L\left(\vec{\theta }\right).$$

To construct the likelihood function we need to make assumptions on the error distribution. There are two types of error: the process error and the observation error. The observation error is the error in the observation process. For example, most people with influenza do not go to see a doctor, and thus there is no record of these cases, resulting in an under-reporting of the number influenza cases. Also, many influenza related deaths are caused by complications such as pneumonia, and influenza may not be recorded as the cause. Typos, miscommunication, etc, can all result in observation errors. The process error originates from the stochasticity of the system that is independent to observation. For example, the disease dynamics is intrinsically stochastic. The time that an infectious individual recovers, and the time that a susceptible individual is infected, are all random variables that affects the number of new infections at any time, even if we eliminate all observation errors. These two types of errors have very different nature, and thus need very different assumptions. For example, it is reasonable to assume that observation errors are independent to each other, but process errors at a later time are commonly dependent on the process errors at earlier times.

### 4.1 Case 1: process errors are negligible

If observation errors are large and process errors are negligible, then we assume that the random variable $X_{i}$ corresponding to the observation $x_{i}$ is independently distributed with a probability mass function $p_{i}\left(k;\vec{\theta }\right)$ where *k* is the values that $X_{i}$ can take. Then, the likelihood function is

$$L\left(\vec{\theta }\right)=\overset{n-1}{\underset{i=0}{\prod }}p_{i}\left(d_{i};\vec{\theta }\right).$$

The maximization of this likelihood function rarely has an analytic solution, and commonly needs to be solved numerically. Note that each factor (probability) can be very small, and thus the product may be very difficult to minimize numerically because of rounding errors (from the binary representation of real numbers in computers). It is a common practice to maximize the log-likelihood function

$$\ell \left(\vec{\theta }\right)=\ln\;L\left(\vec{\theta }\right)=\overset{n}{\underset{i=0}{\sum }}\ln p_{i}\left(d_{i};\vec{\theta }\right).$$

For example, we assume that the number of cases $x\left(t_{i}\right)$ at time $t_{i}$ is independently Poisson distributed with mean $\mu_{i}=c_{0}e^{\lambda t_{i}}$. Then, the log-likelihood function

$$\ell \left(c_{0},\lambda \right)=\overset{n}{\underset{i=0}{\sum }}\ln\frac{e^{-\mu_{i}}\mu_{i}^{x_{i}}}{x_{i}!}=\overset{n-1}{\underset{i=0}{\sum }}-\mu_{i}+x_{i}\;\ln\mu_{i}-\ln x_{i}!.$$

Note that the observed cases $x_{i}$ are constants, and thus the last term can be ignored for maximization. Thus,

$$\left({\hat{c}}_{0},\hat{\lambda }\right)=\underset{\left(c_{0},\lambda \right)}{\text{argmax}}\overset{n-1}{\underset{i=0}{\sum }}-\mu_{i}+x_{i}\;\ln\mu_{i}=\underset{\left(c_{0},\lambda \right)}{\text{argmax}}\overset{n-1}{\underset{i=0}{\sum }}-c_{0}e^{\lambda t_{i}}+x_{i}\;\ln c_{0}+\lambda x_{i}t_{i}.$$

This maximization problem can only be solved numerically.

We choose Poisson distribution because its simple form greatly simplifies the log-likelihood function. In addition, it does not introduce more parameters, which is valuable to avoid over-fitting when the number of data points available is small. If the process error is not completely negligible, then choosing an overly dispersed distribution, such as the negative binomial distribution may be desirable. A negative binomial distribution has two parameters, the success probability q≥0 and the shape parameter r>0. For simplicity, we assume that the shape parameter *r* is the same at each time $t_{i}$, and will; be estimated together with the model parameters $\vec{\theta }$; but *q* depend on $t_{i}$. The probability mass function is

$$p_{i}\left(k;q_{i},r\right)=\frac{\text{Γ}\left(k+r\right)}{k!\text{Γ}\left(r\right)}q_{i}^{k}{\left(1-q_{i}\right)}^{r},$$

with the mean

$$\mu_{i}=\frac{q_{i}r}{1-q_{i}}=c_{o}e^{\lambda t_{i}}.$$

Thus,

$$q_{i}=\frac{c_{0}e^{\lambda t_{i}}}{r+c_{0}e^{\lambda t_{i}}},$$

and the log-likelihood function is

$$\ell \left(c_{0},\lambda ,r\right)=\overset{n-1}{\underset{i=0}{\sum }}\text{ln}\frac{\text{Γ}\left(x_{i}+r\right)}{x_{i}!\text{Γ}\left(r\right)}\frac{c_{0}^{x_{i}}e^{\lambda x_{i}t_{i}}r^{r}}{{\left(r+c_{0}e^{\lambda t_{i}}\right)}^{x_{i}+r}}$$

$$=\overset{n-1}{\underset{i=0}{\sum }}\ln\text{Γ}\left(x_{i}+r\right)-\ln\text{Γ}\left(r\right)+x_{i}c_{0}+\lambda x_{i}t_{i}+r\;\ln\;r-\left(x_{i}+r\right)\ln\left(r+c_{0}e^{\lambda t_{i}}\right)-\ln x_{i}!.$$

Again, the last term can be ignored for the optimization problem. In addition, there is a constraint r>0.

### 4.2 Case 2: observation errors are negligible

If process errors are large and observation errors are negligible, then we cannot assume that the observed values $X_{i+1}$ and $X_{i}$ are independent to each other. Instead, for all i=0,1,…,n−2, we compute the probability mass function of $X_{i+1}$ given ${\left\{X_{j}=x_{j}\right\}}_{j=0}^{i}$, namely, $q_{i+1}\left(k;\vec{\theta }|{\left\{x_{j}\right\}}_{j=0}^{i}\right)$. Then, the likelihood function is

$$L\left(\vec{\theta }\right)=P\left({\left\{x_{i}\right\}}_{i=0}^{n-1}|\vec{\theta }\right)$$

$$=P\left(x_{n-1}|{\left\{x_{i}\right\}}_{i=0}^{n-2},\vec{\theta }\right)P\left({\left\{x_{i}\right\}}_{i=0}^{n-2},\vec{\theta }\right)$$

$$=\overset{n-1}{\underset{i=1}{\prod }}q_{i}\left(x_{i};\vec{\theta }|{\left\{x_{j}\right\}}_{j=0}^{i-1}\right).$$

For simplicity, assume that $X_{i+1}$ is Poisson distribution with mean $\mu_{i+1}=X_{i}e^{\lambda \left(t_{i+1}-t_{i}\right)}$. Note that, since we assumed no observation error, the initial condition $c_{0}=x_{0}$ is exact, and thus there is a single parameter λ for the model. Thus,

$$q_{i+1}\left(k;\vec{\theta }|{\left\{x_{j}\right\}}_{j=0}^{i}\right)=\frac{e^{\mu_{i+1}}\mu_{i+1}^{k}}{k!},$$

and thus the log-likelihood function is

$$l\left(\vec{\theta }\right)=\overset{n-1}{\underset{i=0}{\sum }}\text{ln}\frac{e^{\mu_{i}}\mu_{i}^{x_{i}}}{x_{i}!}$$

$$=\overset{n-1}{\underset{i=0}{\sum }}\mu_{i}+x_{i}\;\ln\mu_{i}-\ln x_{i}!$$

$$=\overset{n-1}{\underset{i=0}{\sum }}x_{i-1}e^{\lambda \left(t_{i}-t_{i-1}\right)}+x_{i}\lambda \left(t_{i}-t_{i-1}\right)+x_{i}\;\ln x_{i}-\ln x_{i}!.$$

Again, the last two terms can be ignored in maximization because they are constants. Thus,

$$\lambda =\underset{\lambda }{\text{argmax}}\;x_{i-1}e^{\lambda \left(t_{i}-t_{i-1}\right)}+\left(t_{i}-t_{i-1}\right)x_{i}\lambda .$$

### 4.3 Case 3: consider both type of errors together

It is much harder to formulate the likelihood function if process errors and observation errors must both be considered. We can simplify the problem by ignoring the process error and use an overly dispersed observation error distribution as a compensation. Note that this simplification mainly affects the confidence intervals.

### 4.4 Confidence intervals

The maximum likelihood method gives a point estimate, i.e., one set of parameter values that makes it mostly likely to observe the data. However, it is not clear how close the point estimates are to the real values. To answer this question we use an interval estimate, commonly known as a confidence interval. A confidence interval with a confidence level α is an interval that has a probability α that contains the true parameter value. A commonly used confidence level is 95%, which originates from a normal distribution. If a random variable *X* is normally distributed with a mean μ and a standard deviation σ, then the probability that X∈[μ−2σ,μ+2σ] is 95%.

The confidence interval can be estimated using the likelihood ratio test [(Bolker, 2008), p.192]. Let $\hat{\vec{\theta }}$ be the point estimate of the parameters. A value $\lambda_{0}$ is in the 95% confidence interval is equivalent to accepting with 95% probability that $\lambda_{0}$ is a possible growth rate. To determine this we fit a nested model by fixing the growth rate $\lambda =\lambda_{0}$, suppose its point estimate is ${\hat{\theta }}_{0}$. We then compute the likelihood ratio

$$\text{Λ}=\frac{L\left({\hat{\theta }}_{0}\right)}{L\left(\hat{\theta }\right)}\;.$$

The Wilks’ theorem (Wilks, 1938) guarantees that, as the sample size becomes large, the statistics $-2\text{lnΛ}=2\left[\ell \left(\hat{\theta }\right)-\ell \left({\hat{\theta }}_{0}\right)\right]$ is $\chi^{2}$ distributed with a degree of freedom 1. We thus can compare −2lnΛ with the 95% quantile of the $\chi^{2}$ distribution and determine if $\lambda_{0}$ should be in the confidence interval or not. We can thus perform a linear search on both sides of the point estimate to determine the boundary of the confidence interval.

## 5 Mechanistic and phenomenological models

We still have not addressed the problem of choosing a fitting window for an exponential model. Recall that the challenge arises because the exponential growth rate of an epidemic decreases with time. Instead of finding heuristic conditions for choosing the fitting window, we circumvent this problem by incorporating the decrease of the exponential growth rate into our model. We have two choices, using either a mechanistic model such as an SIR or SEIR model, or a phenomenological model.

### 5.1 Mechanistic models

Naturally, if we know that a mechanistic model is a good description of the disease dynamics, fitting such a model to the epidemic curve is a good option (see, e.g., (Chowell, Ammon, Hengartner, & Hyman, 2006; Pourabbas, d’Onofrio, & Rafanelli, 2001),). We use an SIR model as an example. For simplicity, we assume that the process error is negligible, and the incidence rate is Poisson distributed with a mean C(t) given by an SIR model (C(t)=βSIN where *N* is the population size). To construct the log-likelihood function, we need to calculate C(t), i.e., numerically solve the SIR model. To do so, we need the transmission rate β. the recovery rate γ, the initial fraction of infectious individuals $I\left(0\right)=I_{0}$ (with the assumption that R(0)=0, $S\left(0\right)=1-I_{0}$, and thus $I_{0}$ determines the initial conditions), in addition to the population size *N*. Thus, the parameters of the model is $\vec{\theta }=\left(\beta ,\gamma ,I_{0},N\right)$. Thus the log-likelihood function is (ignoring the constant terms)

$$\ell \left(\beta ,\gamma ,I_{0},N\right)=\overset{n-1}{\underset{i=0}{\sum }}-c\left(t_{i}\right)+x_{i}\;\ln\;c\left(t_{i}\right)\;,$$

where the number of new cases $c\left(t_{i}\right)$ in the time interval $\left[t_{i},t_{i+1}\right]$ is

$$c\left(t_{i}\right)=S\left(t_{i+1}\right)-S\left(t_{i}\right)\;,$$

and $S\left(t_{i}\right)$ is solved numerically from the SIR model. Thus, ℓ implicitly depend on β, γ and $I_{0}$ through S(t).

One draw back using such a mechanistic model is its high computational cost, since each evaluation of the log-likelihood function requires solving the model numerically, and numerical optimization algorithms can be very hungry on function evaluations, especially if the algorithm depends on numerical differentiation.

Another draw back is that these mechanistic models can be overly simplified, and may not be a good approximation to the real disease dynamics. For example, for seasonal influenza, due to the fast evolution of the influenza virus, individuals have different history of infection, and thus have different susceptibility to a new strain. Yet simple SIR and SEIR models assume a population with a homogeneous susceptibility. Thus using a simple SIR to fit to an influenza epidemic may be an over simplification. However, realistic mechanistic models can be overly complicated, and involve too many parameters that are at best difficult to estimate. For example, a multi-group SIR model depends on a contact matrix consisting of transmission rates between groups, which contains a large number of parameters if the model uses many groups.

### 5.2 Phenomenological models

If all we need to estimate is the exponential growth rate, we only need a model that describes the exponential growth that gradually slows down. Most cumulative epidemic curves grow exponentially initially, and then saturates at the final epidemic size. A simple phenomenological model can be used to describe the shape of the cumulative epidemic curve, but the model itself may not have realistic biological meaning. However, if simple mechanistic models cannot faithfully describe the epidemic process, using a simple phenomenological model with an analytical formula may be a better choice, at least numerically, because repetitively solving a system differential equations numerically, and differentiating the log-likelihood function numerically, can both be avoided with the analytical formula. Here we discuss some examples for such models.

#### Logistic model

The logistic model is the simplest model that shows an initial exponential growth followed a gradual slowing down and a saturation. The cumulative incidences C(t) (the total number of cases by time *t*) can be approximated by

$$\frac{d}{dt}C\left(t\right)=rC\left(t\right)\left(1-\frac{C\left(t\right)}{K}\right).$$

where *r* is the exponential growth rate, and $K={\text{lim}}_{t\to \infty }C\left(t\right)$. Let $C_{0}=C\left(0\right)$, its solution is

(7) $$C\left(t\right)=\frac{KC_{0}}{C_{0}+\left(K-C_{0}\right)e^{-rt}},$$

The new cases $c\left(t_{i}\right)$ in a time period $\left[t_{i},t_{i+1}\right]$ is thus

(8) $$c\left(t_{i}\right)=C\left(t_{i+1}\right)-C\left(t_{i}\right)\;.$$

The model parameters are $\vec{\theta }=\left(r,K,C_{0}\right)$. Note that it is less than the number of parameters of the simplest mechanistic model (i.e., the SIR model).

#### Richards model

The logistic model has a fixed rate of slowing down of the exponential growth rate. To be more flexible, we can use the Richards model (Richards, 1959) for the cumulative incidence curve. The Richards model, also called the power law logistic model, can be written as

$$\frac{d}{dt}C\left(t\right)=rC\left(t\right)\left[1-{\left(\frac{C\left(t\right)}{K}\right)}^{\alpha }\right],$$

where αis the parameter that controls the steepness of the curve. Note that the logistic model is a special case with α=1. Its solution is

$$C\left(t\right)=\frac{KC_{0}}{{\left[C_{0}^{\alpha }+\left(K^{\alpha }-C_{0}^{\alpha }\right)e^{-\frac{rtK^{\alpha }}{K^{\alpha }-C_{0}^{\alpha }}}\right]}^{1/\alpha }}.$$

The new cases $c\left(t_{i}\right)$ in a time period $\left[t_{i},t_{i+1}\right]$ is also given by (8). The parameters are $\vec{\theta }=\left(r,K,C_{0},\alpha \right)$.

### 5.3 Comparison of the models

To compare the performance of both the SIR model and the phenomenological models, we fit these models to the stochastically simulated SEIR epidemic curve of weekly cases that we introduced in Section 3 (Fig. 3).

**Fig. 4:**
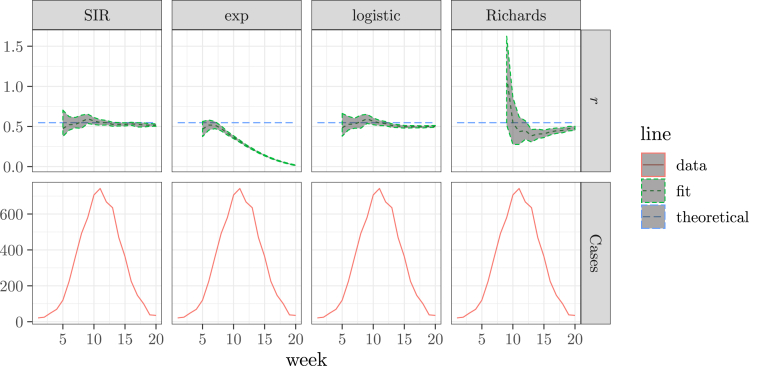
The comparison of the results of fitting the SIR, exponential, logistic, and Richards models to a simulated weekly incidence curve, as a function of the end point of the fitting window (upper). The epidemic curve (lower) is shown as a reference. The epidemic curve and the theoretical exponential growth rate are the same as Fig. 3 s.

We assume that the process error is negligible, and the observations are Poisson distributed about the mean that is given by the corresponding models. We use the maximum likelihood method. The results are shown in Fig. 4. The predictions of the exponential model, as discussed before, quickly decreases as more data points are used. Both the logistic model and the Richards model give robust estimates with fitting windows ending up to the peak of the epidemic. The SIR model gives a robust estimate for all fitting windows up to the whole epidemic curve.

Thus, the SIR model is a good model to use to fit the exponential growth rate, even if it may not be the correct mechanistic model. (e.g., it ignores the latent period in this example). It requires more computational power, because the epidemic curve lacks an analytic formula, and needs to be numerically solved from a system of ordinary differential equations. The logistic model and the Richards model can be used for all data points up to the peak of the epidemic.

### 5.4 Coverage probabilities

Fig. 4 also show that the SIR model and the logistic model give the narrowest confidence intervals. However, narrower confidence intervals may not be desirable if it has a large chance that it does not contain the true value. Due to errors, especially process errors, each realization of the underlying stochastic epidemic process yields a different epidemic curve. These epidemic curves may exhibit different exponential growth rates even if the underlying parameter values are the same. An observed epidemic curve is just a single realization of the epidemic process. Does the estimated confidence intervals contain the theoretical exponential growth rate of the epidemic process? This question is answered by the “coverage probability”, which is the probability that the confidence interval contains the true value. If the confidence interval properly considers all sources of stochasticity, then the coverage probability should be equal to its confidence level.

To illustrate this, we numerically compute the coverage of the confidence intervals by simulating the SEIR model 400 times and compute confident interval of the exponential growth rate for each realization, and compute the fraction of the confident intervals containing the theoretical value λ=0.537. The results is summarized in below:

**Table:** 

	logistic model	Richards model
coverage probability	43%	65%

That is, even though the logistic model gives a narrow confidence interval, its coverage probability is low. The coverage probability of the confidence interval given by the Richards model is also significantly lower than the confidence level. This is indeed caused by treating process errors as observation errors. If there is under reporting, that is, only a fraction *p* of the cases can be observed, then the observation error becomes larger as *p* decreases (i.e., more under reporting). The coverage will become larger as a result. For example, the case fatality ratio of the 1918 pandemic influenza is about 2% (Frost, 1920). Thus, the mortality curve can be treated as the epidemic curve with a large under reporting ratio, and thus the observation error dominates. In this case ignoring the process error is appropriate.
